# PRIMEtime CE: a multistate life table model for estimating the cost-effectiveness of interventions affecting diet and physical activity

**DOI:** 10.1186/s12913-019-4237-4

**Published:** 2019-07-16

**Authors:** Adam D. M. Briggs, Linda J. Cobiac, Jane Wolstenholme, Peter Scarborough

**Affiliations:** 1 Centre on Population Approaches for Non-Communicable Disease Prevention and NIHR Biomedical Research Centre at Oxford, Nuffield Department of Population Health, University of Oxford, Old Road Campus, Headington, Oxford, OX3 7LF UK; 2 Health Economics Research Centre, Nuffield Department of Population Health, University of Oxford, Oxford, UK

**Keywords:** Public health economics, Modelling, Economic modelling, Non-communicable disease, Diet, Physical activity, Public health

## Abstract

**Background:**

Non-communicable diseases are the leading cause of death in England, and poor diet and physical inactivity are two of the principle behavioural risk factors. In the context of increasingly constrained financial resources, decision makers in England need to be able to compare the potential costs and health outcomes of different public health policies aimed at improving these risk factors in order to know where to invest so that they can maximise population health. This paper describes PRIMEtime CE, a multistate life table cost-effectiveness model that can directly compare interventions affecting multiple disease outcomes.

**Methods:**

The multistate life table model, PRIMEtime Cost Effectiveness (PRIMEtime CE), is developed from the Preventable Risk Integrated ModEl (PRIME) and the PRIMEtime model. PRIMEtime CE uses routinely available data to estimate how changing diet and physical activity in England affects morbidity and mortality from heart disease, stroke, diabetes, liver disease, and cancers either directly or via raised blood pressure, cholesterol, and body weight.

**Results:**

Model outcomes are change in quality adjusted life years, and change in English National Health Service and social care costs.

**Conclusion:**

This paper describes PRIMEtime CE and highlights its main strengths and limitations. The model can be used to compare any number of public policies affecting diet and physical activity, allowing decision makers to understand how they can maximise population health with limited financial resources.

**Electronic supplementary material:**

The online version of this article (10.1186/s12913-019-4237-4) contains supplementary material, which is available to authorized users.

## Background

Non-communicable diseases (NCDs) are responsible for 88% of the total disease burden in England, 38% of which is attributable to potentially amenable behavioural, environmental, and metabolic risk factors [1]. The four leading behavioural risk factors for disease in England are tobacco, unhealthy diets, alcohol and drug misuse, and physical inactivity [1]. Of these, poor diet and physical inactivity account for a quarter of the total attributable disease burden, a burden that could be significantly reduced through public health interventions [1–4].

Over recent years, there have been increasing pressures on acute health services in England and as a consequence, health providers are arguing for there to be a greater emphasis placed on prevention. Both the 2019 NHS Long Term Plan and the UK Secretary of State for Health and Social Care’s 2018 vision for prevention explicitly state that prevention and population health improvement are policy priorities [5, 6]. In order for prevention and public health to play their part in maintaining the sustainability of the NHS, public health practitioners and decision makers need to have the information, influence, and resources to make the best decisions about how to spend finite resources.

In 2013, the structure of public health in England changed with public health responsibility moving from the NHS to local government [7]. This allows public health professionals to influence more readily the wider determinants of health whilst also making them vulnerable to local government budget constraints [8]. In 2016 the UK Health Select Committee highlighted the challenges faced by public health professionals, citing evidence that real-terms funding of public health in England will be cut from £3.47bn to just over £3bn between 2015/16 and 2020/21 [9].

Despite shrinking public health budgets, there is still significant potential to improve population health using prevention strategies that may be cost-effective or cost saving [2, 3, 10, 11]. To make informed choices about how to maximize population health with limited resources, local and national decision makers need to quantify and compare the possible impact, cost, and opportunity costs of different interventions.

### Public health economic modelling

There are well established methods for modelling the cost-effectiveness of healthcare interventions. For example, the International Society for Pharmacoeconomics and Outcomes Research-Society for Medical Decision Making (ISPOR-SMDM) guidelines for modelling research have published multiple best practice guidelines [12]. Furthermore, The National Institute for Health and Care Excellence (NICE) has guidance on methods for health economic modelling [13]. The ISPOR-SMDM guidelines and NICE guidance aim to standardise methods for health technology assessments (HTAs) so that the results from different studies can be directly compared with each other against a decision framework, such as the NICE cost effectiveness threshold of £20,000 to £30,000 [13]. However, economic evaluations of public health interventions have their own specific challenges compared with HTAs such as quantifying long term outcomes, wider societal consequences and the impact on inequalities, and the effects of multicomponent interventions [14–24].

In 2005, NICE started producing guidance on public health interventions, including economic modelling [13, 25, 26]. The NICE reference case now includes advice on how to address some of the challenges of public health economic evaluations: for example, the time horizon should be long enough to incorporate all important costs and effects; the perspective on costs may be public sector, societal, or any other as appropriate; and non-health benefits to local government and other settings may also be included [13]. However, the guidance is not prescriptive, meaning that different public health economic modellers often use different model structures, time horizons, health and economic perspectives, epidemiological data, and outcome measures [24]. Even within academic fields with a well-established history of health economic modelling, such as diabetes, chronic obstructive pulmonary disease (COPD), and cancer, different model structures and assumptions can produce very different outcomes despite modelling the same intervention [27–29]. Therefore, often it is not possible to compare results directly when prioritising different public health policies. As such, standardised processes are required for assessing and modelling the cost, health impact, and cost-effectiveness of public health interventions.

### Aim

In this paper we describe the PRIMEtime Cost Effectiveness (PRIMEtime CE) model. PRIMEtime CE addresses some of the challenges outlined above by being able to directly compare the cost-effectiveness of public health policies aimed at improving population diet or levels of physical activity.

## Methods

PRIMEtime CE estimates the cost-effectiveness and return on investment of interventions affecting the population distribution of physical activity levels and 13 dietary risk factors in a modelled population. Modelled interventions can affect risk factors either in isolation or in any given combination.

PRIMEtime CE was developed from the Preventable Risk Integrated ModEl (PRIME) and the PRIMEtime model. PRIME is a cross-sectional comparative risk assessment model that estimates the age and sex-specific impact on NCD mortality from changing the population distribution of 10 dietary risk factors, plus physical activity, smoking, and alcohol. Modelled diseases include seven types of cardiovascular disease, diabetes, 13 cancer subtypes, chronic obstructive pulmonary disease, kidney disease, and liver disease. Each parameter used in the model is drawn from a meta-analysis of either prospective cohort studies or randomised controlled trials. The statistical detail underlying PRIME has been previously published, including listing various publications arising from the model [30].

### PRIMEtime

The PRIMEtime model is a multistate life table model which quantifies the effect of changing 14 dietary risk factors (the 10 risk factors included in PRIME plus processed meat, red meat, free sugars, and fibre from cereals) on morbidity and mortality from ischaemic heart disease (IHD), type two diabetes, stroke, seven cancer subtypes (breast, lung, colorectal, stomach, liver, kidney, and pancreas), and liver cirrhosis in the UK population [31]. The model combines the dietary risk factors included in the PRIME model [30] with multistate life table methods developed by Cobiac and colleagues in Australia and New Zealand [32–34]. It simulates a closed adult population cohort (aged 15 and above) by single year of age and by sex over the lifetime of the cohort or until individuals reach 100 years of age. It uses UK specific data by age and sex (where available) on baseline disease incidence, prevalence, case-fatality rates (the annual mortality rate among prevalent cases), and disease trends.

As with PRIME, the relationships between diet and disease are parameterised using meta-analyses of randomised controlled trials or prospective observational studies and are modelled either as direct effects or via one of three intermediate risk factors: blood pressure, BMI, and total cholesterol. PRIMEtime is built in Microsoft Excel and uses Ersatz and EpiGearXL add-ins from EpiGear International to run Monte Carlo probabilistic sensitivity analyses and quantify the uncertainty in model input data [35–37]. The 2.5th and 97.5th percentiles of multiple model runs are used to estimate 95% uncertainty intervals (UIs, usually 2000 runs by which point uncertainty ranges have converged on a stable value). Tables 1, 2, 3, 4, 5 and 6 show the sources of input data and uncertainty distributions included in PRIMEtime and PRIMEtime CE.

**Table 1:** Disease specific data inputs for PRIMEtime CE (all age and sex specific), reproduction of table 1 in supplementary file of Cobiac et al. (with permission) [31]

Disease	Data and methods
Coronary heart disease (CHD)	Incidence of CHD estimated from incidence rates of first acute myocardial infarction (derived from Hospital Episode Statistics [38]), adjusted using the proportion of unstable angina among all coronary events in the OXVASC study [39]. Mortality rates from the Office of National Statistics cause-specific death registrations (number of deaths where myocardial infarction was mentioned on the death certificate) [40]. Case fatality rates and baseline prevalence derived using DISMOD II^a^ [41].
Stroke	Incidence of first stroke estimated from the OXVASC study [39] and data from the General Practice Research Database [42]. Mortality rates from the Office of National Statistics cause-specific death registrations [40]. Case fatality rates and baseline prevalence derived using DISMOD II [41].
Type two diabetes	Incidence rates from the UK Clinical Practice Research Datalink [43]. Type two diabetes mortality rate ratios and prevalence estimated from the National Diabetes Audit 2011/12 [44]. Case fatality rates derived using DISMOD II [41].
Cirrhosis	Incidence rates from a population-based cohort study linking the Clinical Practice Research Datalink and Hospital Episode Statistics [45]. Mortality rates from the Office of National Statistics cause-specific death registrations [40]. Case fatality rates and baseline prevalence derived using DISMOD II [41].
Cancers	Incidence rates from Cancer Registrations Statistics, England, 2012 [46]. Mortality rates from the Office of National Statistics cause-specific death registrations [40]. Case fatality rates and baseline prevalence derived using DISMOD II [41].

NB Background UK disease trends derived by Cobiac et al. using methodology from the global burden of disease project [31, 47, 48].

^a^Case fatality refers to the annual mortality rate among prevalent cases

**Table 2:** PRIMEtime CE risk factors directly related to disease, exposure parameters, outcomes, and modelled uncertainty distributions. Adapted and updated from Cobiac et al.[32] Dietary relative risks adjusted for energy intake where possible

Risk factors						
Parameter	Exposure parameters	Outcomes	Unit of change	Distribution of uncertainty range	Relative risk (SE)	Notes
Fruit	Mean (SD) g/day for consumers and % consuming < 1 fruit portion daily from the National Diet and Nutrition Survey (NDNS) [49]. Theoretical ideal: 300 (30) g/day [50]	CHD [51]	Per 106 g/day fruit	Lognormal	0.93 (0.019)	
		Stroke [52]	Per 106 g/day fruit	Lognormal	0.89 (0.023)	
		Lung cancer [53]	Per 100 g/day fruit	Lognormal	0.94 (0.02)	
Vegetables	Mean (SD) g/day for consumers and % consuming < 1 vegetable portion daily from NDNS [49]. Theoretical ideal: 400 (30) g/day [50]	CHD [51]	Per 106 g/day vegetables	Lognormal	0.89 (0.034)	
		Lung cancer [53]	Per 100 g/day vegetables	Lognormal	0.94 (0.025)	
Fibre (cereal only)	Mean (SD) g/day from NDNS [49]	CHD [54]	Per 10 g/day fibre (cereal)	Lognormal	0.91 (0.02)	Cereals only to avoid double counting for fruit and vegetables
Fibre	Mean (SD) g/day from NDNS [49]. Theoretical ideal: 30 (3) g/ day [50]	Breast cancer [55]	Per 10 g/day fibre	Lognormal	0.93 (0.027)	
		Colorectal cancer [56]	Per 10 g/day fibre	Lognormal	0.90 (0.034)	
		Stomach cancer [57]	Per 10 g/day fibre	Lognormal	0.56 (0.12)	
Red meat intake	Mean (SD) g/day from NDNS [49]. Theoretical ideal: 100 (10) g/ week [50]	Colorectal cancer [58]	Per 100 g/day red meat	Lognormal	1.30 (0.06)	
		Stomach cancer [59]	Per 100 g/day red meat	Lognormal	1.13 (0.036)	
		Type two diabetes [60]	Per 100 g/day red meat	Lognormal	1.20 (0.072)	
Processed meat intake	Mean (SD) g/day from NDNS [49]. Theoretical ideal: 0 g/day [50]	Colorectal cancer [58]	Per 50 g/day processed meat	Lognormal	1.38 (0.07)	
		Type two diabetes [60]	Per 50 g/day processed meat	Lognormal	1.57 (0.1)	
Serum cholesterol	Mean (SD) mmol/L from NDNS [49]. Theoretical ideal: 3.8 (0.6) mmol/L [61]	CHD [62]	Per -1 mmol/l total cholesterol	Lognormal	< 49 years: 0.44 (0.034) 50–59 years: 0.58 (0.034) 60–69 years: 0.72 (0.018) 70–79 years: 0.82 (0.015) 80+ years: 0.85 (0.21)	
		Stroke [62]	Per -1 mmol/l total cholesterol	Lognormal	< 59 years: 0.90 (0.037) 60–69 years: 1.02 (0.027) 70–79 years: 1.04 (0.025) 80+ years: 1.06 (0.031)	
Systolic blood pressure	Mean (SD) mmHg from NDNS [49]. Theoretical ideal: 115 (6) mmHg [61]	CHD [63]	Per -20 mmHg systolic blood pressure	Lognormal	< 49 years: 0.49 (0.042) 50–59 years: 0.50 (0.015) 60–69 years: 0.54 (0.0094) 70–79 years: 0.60 (0.013) 80+ years: 0.67 (0.023)	
		Stroke [63]	Per -20 mmHg systolic blood pressure	Lognormal	< 49 years: 0.36 (0.057) 50–59 years: 0.38 (0.034) 60–69 years: 0.43 (0.024) 70–79 years: 0.50 (0.02) 80+ years: 0.67 (0.03)	
Body mass index	Mean (SD) kg/m^2^ from NDNS [49]. Theoretical ideal: 21 (1) kg/m2 [61]	CHD [64]	Per 5 kg/m^2^ BMI	Lognormal	35–59 years: 1.50 (0.039) 60–69 years: 1.40 (0.031) 70–79 years: 1.31 (0.033) 80–89 years: 1.30 (0.055)	
		Stroke [64]	Per 5 kg/m^2^ BMI	Lognormal	35–59 years: 1.76 (0.075) 60–69 years: 1.49 (0.056) 70–79 years: 1.33 (0.056) 80–89 years: 1.10 (0.083)	
		Diabetes [64]	Per 5 kg/m^2^ BMI	Lognormal	BMI 15–25: 0.96 (0.25) BMI 25–50: 2.16 (0.067)	
		Pancreas cancer [65]	Per 5 kg/m^2^ BMI	Lognormal	1.10 (0.016)	
		Colorectal cancer [64]	Per 5 kg/m^2^ BMI	Lognormal	Men: 1.24 (0.016) Women: 1.09 (0.019)	
		Breast cancer [64]	Per 5 kg/m^2^ BMI	Lognormal	Women 60+ years: 1.12 (0.018)	
		Kidney cancer [64]	Per 5 kg/m^2^ BMI	Lognormal	Men: 1.24 (0.039) Women: 1.34 (0.034)	
		Liver cancer [64]	Per 5 kg/m^2^ BMI	Lognormal	1.47 (0.078)	
		Liver cirrhosis [64]	Per 5 kg/m^2^ BMI	Lognormal	BMI 15–25: 0.73 (0.016) BMI 25–50: 1.79 (0.077)	
Diabetes	Prevalence	CHD [66]	If have diabetes compared to no diabetes	Lognormal	Men: 1.85 (0.063) Women: 2.63 (0.076)	
		Stroke [67]	If have diabetes compared to no diabetes	Lognormal	Men: 1.83 (0.067) Women: 2.28 (0.085)	
Physical activity	Mean (SD) MET hours per week from Active People’s Survey [68]	CHD [69]	11.25METhr/wk. increase	Normal	−0.204 (0.027)	Parameter is the beta value of the 0.25 transformation of the relative risk, unadjusted for BMI.
		Stroke [69]	11.25METhr/wk. increase	Normal	−0.195 (0.041)	Parameter is the beta value of the 0.25 transformation of the relative risk, unadjusted for BMI.
		Diabetes [69]	11.25METhr/wk. increase	Normal	−0.240 (0.023)	Parameter is the beta value of the 0.25 transformation of the relative risk, unadjusted for BMI.
		Colorectal cancer [70]	11.25METhr/wk. increase	Normal	−0.080 (0.059)	Parameter is the beta value of the 0.25 transformation of the relative risk, unadjusted for BMI.
		Breast cancer [70]	11.25METhr/wk. increase	Normal	−0.053 (0.023)	Parameter is the beta value of the 0.25 transformation of the relative risk, unadjusted for BMI.

*SD* standard deviation, *NDNS* National Diet and Nutrition Survey, *CHD* coronary heart disease, *BMI* body mass index, *MET* Metabolic Equivalent of Task

**Table 3:** PRIMEtime CE risk factors operating through intermediate variables, exposure parameters, outcomes, and modelled uncertainty distributions. Adapted and updated from Cobiac et al.[32] Dietary relative risks adjusted for energy intake where possible

Risk factors operating through intermediate variables						
Parameter	Exposure parameters	Outcomes	Unit of change	Distribution of uncertainty range	Change in value of outcome (SE)	Notes
Total fat	% of total energy [49]	Total serum cholesterol (mmol/l) [71]	Per 1% energy total fat	Normal	0.020 (0.005)	
Saturated fat	% of total energy [49]	Total serum cholesterol (mmol/l) [71]	Per 1% energy saturated fat	Normal	0.052 (0.003)	
Monounsaturated fatty acids	% of total energy [49]	Total serum cholesterol (mmol/l) [71]	Per 1% energy MUFA	Normal	0.005 (0.003)	
Polyunsaturated fatty acids	% of total energy [49]	Total serum cholesterol (mmol/l) [71]	Per 1% energy PUFA	Normal	−0.026 (0.004)	
Dietary cholesterol	mg/day [49]	Total serum cholesterol (mmol/l) [71]	Per 1 g/day dietary cholesterol	Normal	0.0007 (0.0001)	
Salt consumption	g/day [49]	Systolic blood pressure (mmHg) [72]	Per 100 mmol/24 h urinary sodium	Normal	5.80 (1.71)	Grams of salt consumed per day converted into urinary sodium excretion.
Total energy	kJ/day [49]	BMI (kg/m^2^) [73]				Details of equations describing the relationship between energy intake and body weight for men and women can be found in Christiansen and Garby [74].

*SD* standard deviation, *NDNS* National Diet and Nutrition Survey, *CHD* coronary heart disease, *BMI* body mass index, *MET* Metabolic Equivalent of Task

**Table 4:** PRIMEtime CE risk factors' theoretical minimum risk and modelled uncertainty distributions. Adapted and updated from Cobiac et al.[32]

Risk factors’ theoretical minimum risk						
Parameter	Exposure parameters	Outcomes	Unit of change	Distribution of uncertainty range	Minimum risk value (SE)	Notes
Body mass index (kg/m^2^) [61]	–	–	–	Normal	21 (1)	
Systolic blood pressure (mmHg) [61]	–	–	–	Normal	115 (6)	
Total cholesterol (mmol/L) [61]	–	–	–	Normal	3.8 (0.6)	
Vegetable intake (g/day) [50]	–	–	–	Normal	400 (30)	
Fruit intake (g/day) [50]	–	–	–	Normal	300 (30)	
Fibre intake (g/day) [50]	–	–	–	Normal	30 (3)	
Red meat intake (g/day) [50]	–	–	–	Normal	14.3 (1.43)	
Processed meat intake (g/day) [50]	–	–	–	Normal	0	
Physical activity (METhrs/wk) [74]	–	–	–	Normal	133 (13.3)	

*SD* standard deviation, *NDNS* National Diet and Nutrition Survey, *CHD* coronary heart disease, *BMI* body mass index, *MET* Metabolic Equivalent of Task

**Table 5:** PRIMEtime CE mediation factors, exposure parameters, outcomes, and modelled uncertainty distributions. Adapted and updated from Cobiac et al.[32]

Mediation factors						
Parameter	Exposure parameters	Outcomes	Unit of change	Distribution of uncertainty range	Change in value of outcome (SE)	Notes
Ischaemic stroke mediation	BMI (kg/m^2^)	Systolic blood pressure (mmHg) [74]		Normal	0.65 (0.04)	
	Fruit intake (g/day)	Systolic blood pressure (mmHg) [74]		Normal	0.42 (0.17	
	Vegetable intake (g/day)	Systolic blood pressure (mmHg) [74]		Normal	0.54 (0.2)	
	Fruit intake (g/day)	Total cholesterol (mmol/L) [74]		Normal	0.027 (0.017)	
	Vegetable intake (g/day)	Total cholesterol (mmol/L) [74]		Normal	0.047 (0.026)	
Ischaemic heart disease mediation	BMI (kg/m^2^)	Systolic blood pressure (mmHg) [74]		Normal	0.31 (0.016)	
	Fruit intake (g/day)	Systolic blood pressure (mmHg) [74]		Normal	0.39 (0.15)	
	Vegetable intake (g/day)	Systolic blood pressure (mmHg) [74]		Normal	0.47 (0.21)	
	Fruit intake (g/day)	Total cholesterol (mmol/L) [74]		Normal	0.008 (0.0057)	
	Vegetable intake (g/day)	Total cholesterol (mmol/L) [74]		Normal	0.012 (0.01)	

*SD* standard deviation, *NDNS* National Diet and Nutrition Survey, *CHD* coronary heart disease, *BMI* body mass index, *MET* Metabolic Equivalent of Task

**Table 6:** PRIMEtime CE sources and uncertainty distributions for baseline population data, costs, and utilities. All inputs are age and sex specific

Parameter	Data and methods
English population	From Office for National Statistics census data, no uncertainty estimated [75].
Mortality rates	Extracted from the Human Mortality Database, no uncertainty estimated [76].
Health sector costs	Disease specific costs derived from NHS England programme budgeting data [77] and unrelated disease costs estimated using NHS England cost curves [78]. Using the same approach as Blakely et al., [34] health sector costs are assumed to be “moderately uncertain”, and therefore uncertainty is estimated using a generic multiplication factor across all health sector costs with a gamma distribution based on a normal distribution (mean 1, SD 0.1).
Societal costs	Disease specific and unrelated productivity, social care, and wider societal costs estimated using a Department of Health tool published as a supplementary file in Claxton et al [79]. As with health sector costs, uncertainty estimated using a generic multiplication factor across all societal costs with a gamma distribution based on a normal distribution (mean 1, SD 0.1).
Utilities	Baseline mean EQ-5D utility scores and disease specific decrements and their standard errors taken from Sullivan et al., with adjustments made for age and number of chronic conditions [80].

### PRIMEtime CE

PRIMEtime CE is developed from PRIMEtime by adding healthcare and social care costs, estimates of morbidity based on age, sex, and disease state, and physical activity as a behavioural risk factor. The relationship between free sugars and total cholesterol is currently not included in PRIMEtime CE, although this could be added.

**Fig. 1:**
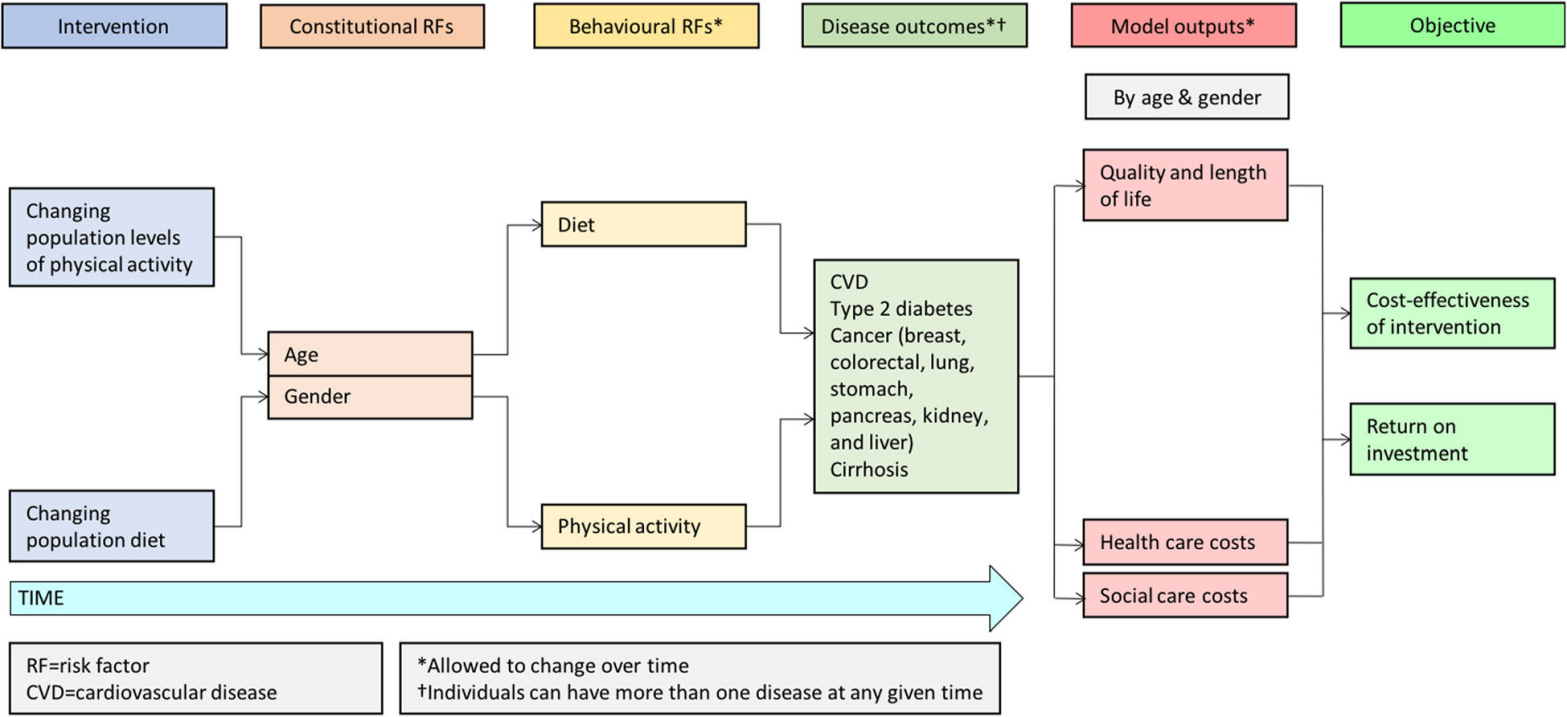
The PRIMEtime CE conceptual model

The conceptual modelling framework for public health economic models published by Squires’ et al. was used to guide the development of a conceptual model [81]. The initial conceptual model was shared with multiple stakeholders who provided feedback on the proposed model outcomes, the relationships described, and its face validity. Stakeholders included national governmental organizations, local governmental organizations, charitable organizations, health professional and academic organizations, and patients and public (see Table 7). The final conceptual model is shown in Fig. 1.

**Table 7:** List of stakeholders

Stakeholder category	Stakeholder
(i) National governmental organisations	National government^a,b^
	Department of Health^a,b^
	National Institute for Health and Care Excellence^a,b^
	Public Health England^b^
	Physical activity and diet responsibility deal^b^
(ii) Local governmental organisations	Local Government Association^b^
	Thames Valley Public Health England Centre^b^
	Oxfordshire County Council Public Health Department^b^
(iii) Charitable organisations	The Wellcome Trust (project funder)
	UK Health Forum^a,b^
	British Heart Foundation^a,b^
	Food Ethics Council^a,b^
	Consensus Action on Salt and Hypertension / Action on Sugar^a,b^
	World Obesity Federation^a,b^
	Diabetes UK^a,b^
	Sustain^a,b^
	Blood Pressure UK^a,b^
(iv) Health professional and academic organisations	Association of Directors of Public Health^b^
	Academy of Medical Royal Colleges^a,b^
	Faculty of Public Health^a,b^
	International Society for Physical Activity and Health^b^
	International Society of Behavioural Nutrition and Physical Activity^b^
	The Nutrition Society^a,b^
	Association for the Study of Obesity^a,b^
(v) Patients and the public	Members of the public^a^
	Patients with chronic disease^a^

^a^contacted to identify scenarios to test; ^b^contacted to get feedback on model structure

### Adding physical activity as a risk factor

Population physical activity levels by age and sex are taken from the Active People Survey, 2010–2011 (APS) [68]. The APS collects information on sport participation rates from a representative English population sample and includes the degree of participation in a variety of activities. Physical activity data from APS were used to calculate the total number of metabolic equivalent of task (MET) minutes per week for each respondent. A MET is defined as consuming 3.5 ml of oxygen per kg of body weight per minute, the resting metabolic rate. Levels of physical exertion can be measured in relation to this resting metabolic rate, for example running at seven miles per hour is equivalent to 11 METs [82]. The APS includes data for 166,275 adults aged 16 years and over after outliers reporting over 200 MET hours/week were removed.

The relationships between physical activity and IHD, stroke, type two diabetes, breast cancer, and colorectal cancer are included in PRIMEtime CE. The beta-coefficient describing the dose-response relationship between physical activity and disease was derived using two recent meta-analyses of observational studies, both conducted by the same research group using the same methodology [69, 70]. The parameters are unadjusted for obesity as this is assumed to act on the causal pathway (the effect of physical activity on disease is modelled directly rather than via BMI).

Each age and sex group in the population is divided into four categories: sedentary (zero minutes of moderate physical activity per week), under active (less than 60 min of moderate physical activity per week but not sedentary), active (60–150 min per week), and recommended (meeting the UK Chief Medical Officers’ recommendations of 150 min of moderate physical activity per week or more) [83]. Examples of moderate physical activity include brisk walking and cycling. The mean and standard error MET hours/week is calculated from APS for each age and sex group in each physical activity category. These can then be applied to results from the meta-analyses to estimate the relative risk of disease compared to being sedentary. These relative risks are used in PRIMEtime CE to predict the change in disease burden following an intervention.

Furthermore, in PRIMEtime CE, decreasing the prevalence of type two diabetes reduces the risk of the modelled cohort developing IHD and stroke. Using the relationships describing the effect of physical activity on IHD, stroke, and diabetes derived using Wahid et al. would over-estimate the effect of physical activity on IHD and stroke as falls in diabetes would result in additional CVD reductions beyond those estimated in the meta-analyses. Therefore, the relative risk of physical activity on IHD and stroke from Wahid et al. was adjusted downwards using results from Cobiac and Scarborough such that the overall effect of physical activity on CVD is the sum of the direct effect plus that mediated via diabetes [84].

Uncertainty in the size of the relationship between physical activity and disease (the beta-coefficient) was estimated from 95% confidence intervals reported in the meta-analyses. Both this uncertainty and uncertainty in the mean physical activity level for each age and sex group based on the standard error reported in APS are included in PRIMEtime CE.

A limitation of this method is that Wahid et al. include domains of physical activity such as occupational activity and household activity that are not captured in the APS. Therefore, the disease relative risks used by PRIMEtime CE are based on higher levels of physical activity than those reported by the APS, where only leisure time physical activity is recorded. As a result, the overall health benefit modelled by PRIMEtime CE may be an over-estimate because the effect of physical activity on health is non-linear with greater health benefits accruing from gains at lower baseline physical activity levels.

### Estimating healthcare and social care costs

Annual disease specific NHS England costs per prevalent case that are comparable between different diseases are derived from 2013/14 programme budgeting data, [77] detailed methods and model costs can be found in Briggs et al [85]. In summary, a macro top-down approach was taken where total NHS England expenditure in 2013/14 is disaggregated to the level of ICD-10 code for diseases included in PRIMEtime CE. Total disease specific costs were divided by the estimated 2014 prevalence of each individual disease in PRIMEtime CE to give the annual cost per prevalent case, averaged across the entire population irrespective of time since diagnosis. The remaining NHS England budget not accounted for by diseases included in PRIMEtime CE was used to calculate the age and sex specific annual healthcare spend on diseases unrelated to those explicitly modelled by PRIMEtime CE that accrues as people age. A summary of included model costs is shown in Table 8.

**Table 8:** Total 2013/14 expenditure by modelled disease (£000 s except cost per prevalent case) Reproduction of Tables 2, 3, 4 and 5 in Briggs et al. (with permission) [85]

Modelled disease	Programme budgeting category	Programme budgeting expenditure	Specialised services expenditure	Primary care expenditure	Total NHS England disease costs	Annual cost per prevalent case (£)
		Step 1	Step 2	Step 3		
Ischaemic heart disease	10a Coronary Heart Disease	953,743	41,818	485,056	1,480,617	1905
Stroke	10b Cerebrovascular disease	689,876	55,443	29,475	774,794	843
Type two diabetes	04a Diabetes	1,071,537	25,577	959,716	2,056,831	444
Breast cancer	02f Cancer, breast	472,192^a^	N/A	N/A	472,192	573
Colon cancer	02c Cancer, lower GI	248,315	84,919	20	333,253	810
Lung cancer	02d Cancer, lung	98,250	33,599	0^b^	131,849	904
Stomach cancer	02b Cancer, upper GI	32,794	11,215	0^b^	44,008	535
Liver cancer	02b Cancer, upper GI	16,990	5810	0^b^	22,801	1532
Kidney cancer	02 h Cancer, urological	25,145	8599	7833	41,577	618
Pancreatic cancer	02b Cancer, upper GI	42,133	14,409	0^b^	56,542	3074
Liver disease	13c Hepatobiliary	59,702	4543	2963	67,209	314

^a^Breast cancer costs are not estimated from programme budgeting expenditure but directly from Luengo-Fernandez et al [86]. *GI* gastrointestinal, *N/A* not applicable; ^b^primary care costs estimated to be negligible

Social care costs are estimated using the wider societal costs tool developed by the Department of Health [78]. The tool estimates the age and sex specific effect on production (paid and unpaid) and consumption following a change in quality of life (quantified using utility values measured by the EQ-5D-3L questionnaire) and ICD-10 code. Consumption includes social care (described as formal care by the tool), informal care (care provided by family and friends), private paid (goods and services purchased for consumption), private unpaid (benefit from goods and services not paid for, such as domestic work), and government (services provided by the government not included in other categories). Social care costs are estimated as a function of age and quality of life, in PRIMEtime CE they are included from the age of 75 years (as per the Department of Health tool) and assume an average monthly cost of £4826 per person (the 2013/14 monthly local authority residential care cost [87]) [79]. This reflects the total societal costs of adult social care rather than the direct costs to local authorities because many people are required to fund a proportion of their care from personal savings.[88]

In PRIMEtime CE, both the change in social care costs arising as a result of changes to modelled diseases and from unrelated diseases due to increasing longevity are included. Age and sex specific utility values for quality of life at baseline and for each disease are calculated using the methods described under ‘Estimating utilities’ below. Productivity gains and wider societal costs can be readily included as sensitivity analyses. A key assumption underlying the Department of Health wider societal costs tool is that quality of life is the key driver of costs, irrespective of diagnosis (stroke and dementia are exceptions to this). In reality this may underestimate the effect of certain diagnoses on an individual’s ability to care for themselves or to be productive.

### Estimating utilities

Morbidity is estimated using Sullivan et al.’s catalogue of EQ-5D utility values [80, 89]. The EQ-5D results were collected by Sullivan et al. from annual US medical expenditure surveys between 2000 and 2003, and included 79,522 unique responses with linked clinical data for the preceding year covering 135 ICD-9 codes; an equivalent representative UK source of EQ-5D results does not exist. The EQ-5D survey results were then valued using time trade off methods by a representative UK population sample (as recommended by NICE) [90].

Matching the ICD-9 disease codes published by Sullivan et al. to ICD-10 codes used by PRIMEtime CE was done with the website www.icd10data.com [91]. The calculation of baseline utility values by age and sex, and disease specific utility decrements followed guidance published by Sullivan et al. in their UK and US papers [80, 92]. To use these utility values to estimate the change in quality adjusted life years (QALYs) in PRIMEtime CE following an intervention, the age and sex specific utility values change with age based on number of years that the model has been running for, and on changes to the proportion of the population in different disease states. If thought important, as with other input parameters to PRIMEtime CE, it is possible to change the utility decrements used within the model either for a primary analysis or for sensitivity analyses. Baseline age and sex specific utility values and the disease specific utility decrements used in PRIMEtime CE are presented in Tables 9 and 10.

**Table 9:** Baseline EQ-5D utility values by age and sex for use in PRIMEtime CE

Age	Male	Female	Age	Male	Female
0	1.000	1.000	51	0.800	0.799
1	1.000	1.000	52	0.800	0.799
2	1.000	1.000	53	0.799	0.798
3	1.000	1.000	54	0.799	0.798
4	1.000	1.000	55	0.799	0.798
5	1.000	1.000	56	0.799	0.798
6	1.000	1.000	57	0.798	0.797
7	1.000	1.000	58	0.798	0.797
8	1.000	1.000	59	0.798	0.797
9	1.000	1.000	60	0.776	0.775
10	0.916	0.916	61	0.776	0.775
11	0.916	0.916	62	0.776	0.775
12	0.916	0.916	63	0.775	0.774
13	0.916	0.916	64	0.775	0.774
14	0.915	0.915	65	0.775	0.774
15	0.915	0.915	66	0.774	0.773
16	0.915	0.915	67	0.774	0.773
17	0.914	0.914	68	0.774	0.773
18	0.914	0.914	69	0.774	0.773
19	0.914	0.914	70	0.725	0.724
20	0.907	0.906	71	0.725	0.724
21	0.907	0.906	72	0.725	0.724
22	0.907	0.906	73	0.724	0.723
23	0.906	0.905	74	0.724	0.723
24	0.906	0.905	75	0.724	0.723
25	0.906	0.905	76	0.724	0.723
26	0.906	0.905	77	0.723	0.722
27	0.905	0.904	78	0.723	0.722
28	0.905	0.904	79	0.723	0.722
29	0.905	0.904	80	0.659	0.658
30	0.881	0.880	81	0.659	0.658
31	0.881	0.880	82	0.658	0.657
32	0.881	0.880	83	0.658	0.657
33	0.880	0.879	84	0.658	0.657
34	0.880	0.879	85	0.658	0.657
35	0.880	0.879	86	0.657	0.656
36	0.880	0.879	87	0.657	0.656
37	0.879	0.878	88	0.657	0.656
38	0.879	0.878	89	0.656	0.655
39	0.879	0.878	90	0.656	0.655
40	0.839	0.838	91	0.656	0.655
41	0.839	0.838	92	0.656	0.655
42	0.839	0.838	93	0.655	0.654
43	0.838	0.837	94	0.655	0.654
44	0.838	0.837	95	0.655	0.654
45	0.838	0.837	96	0.655	0.654
46	0.838	0.837	97	0.654	0.653
47	0.837	0.836	98	0.654	0.653
48	0.837	0.836	99	0.654	0.653
49	0.837	0.836	100	0.653	0.652
50	0.800	0.799

**Table 10:** Disease specific utility values used in PRIMEtime CE

PRIMEtime CE disease outcome	PRIMEtime CE ICD-10 codes	Equivalent ICD-9 codes	Available utility decrement from Sullivan et al. [80]^a^	Utility value used in PRIMEtime CE (SE)
Ischaemic heart disease	I20-I25	410–414	410: −0.063 411: −0.087 412: −0.037 413: −0.085 414: −0.063	Incident case: −0.071 (0.024) Prevalent: −0.070 (0.015)
Stroke	I60-I69	430–438	433: −0.035 435: − 0.033 436: − 0.117 437: − 0.031 438: − 0.073	Incident: − 0.094 (0.019) Prevalent: − 0.046 (0.031)
Type two diabetes	E11, E14	250.×0	250: − 0.071	− 0.071 (0.005)
Breast cancer	C50	174, 175	174: − 0.019	− 0.019 (0.014)
Colon cancer	C18-C20	153, 154.0, 154.1	153: − 0.067	− 0.067 (0.017)
Lung cancer	C34	162.2–162.9	162: − 0.119	−0.119 (0.043)
Stomach cancer	C16	151	151: −0.071	−0.071 (0.105)
Liver cancer	C22	155	155: −0.093	−0.093 (0.044)
Kidney cancer	C64	189.0	189: −0.048	−0.048 (0.041)
Pancreatic cancer	C25	157	195: −0.086	−0.086 (0.027)
Liver disease	K70, K74	571.0–571.3, 571.5, 571.6, 571.9	571: −0.083	−0.083 (0.031)

^a^reported utility decrement controlled for age, comorbidity, gender, race, ethnicity, income, and education; *SE* standard error

The additional change in utility decrement based on an individual’s number of chronic conditions (as estimated by Sullivan et al.) was not included in PRIMEtime CE because of the inability to accurately estimate the baseline prevalence of co-morbidity and how it changes following an intervention. To test the impact of this limitation on results, the model was run firstly using a theoretical scenario without any decrement arising from co-morbidity, and secondly, assuming that everybody had the maximum possible additional decrement. Results were not significantly different from one another.

Sullivan et al. EQ-5D scores are sampled from a non-English patient population survey and there is likely to be underrepresentation of those at very early and late stages of disease as these population groups may be too unwell or unwilling to participate. In order to identify whether utility values published in the Sullivan et al. catalogue were similar to those identified in other populations using different methods, we compared Sullivan et al. with EQ-5D derived breast cancer utility values that were systematically identified from the literature. We used a pre-defined protocol following NICE guidance (see Additional file 1 for protocol) [90].

The systematic review identified 196 studies for full text review from which 23 studies were included for data-extraction. Four studies received the joint highest study *quality and applicability score* (see Additional file 1 for scoring method) and the extracted utility values from these four studies are shown in the Additional file 1: Table S1. The utility values from each of these four studies overlap with the mean utility value among breast cancer patients reported by Sullivan et al [80].

## Results

### Estimating the effects and costs of the interventions and model validation

Interventions affecting any of the risk factors included in PRIMEtime CE can be modelled based on the intervention in question. Examples of this and a discussion of the model’s validation can be found in Briggs et al [93].

Following stakeholder feedback, the primary outcomes of PRIMEtime CE are cost-effectiveness and return on investment (for interventions that are cost-saving) from an English health and social care perspective over a 10-year time horizon. Cost-effectiveness is calculated as: (C_b_ – C_a_) / (E_b_ – E_a_); where C_b_ is the sum of intervention costs and expenditure on health and social care in the 10 years following the intervention; C_a_ is the 10 year costs of health and social care in the scenario where there is no intervention; E_b_ is the total number of QALYs experienced by the modelled population in the 10 years following the intervention; and E_a_ is the same but where no intervention is modelled. In the case where (C_b_ – C_a_) is negative and therefore the intervention is cost saving compared with no intervention, return on investment is the money saved for every £1 spent: (C_b_ – C_a_) / C_i_; where C_i_ is the cost of the intervention. Included are all costs of the intervention, whether they are incurred by government organisations such as the NHS and local authorities, or by industry where appropriate, as these costs may be relevant to decision makers. Costs and health outcomes are discounted at 1.5% as recommended by NICE for interventions likely to have long-term health benefits [13] (see Table 11). However, there is flexibility within the PRIMEtime CE model to manipulate all of these parameters to either change the model’s primary outcomes, or as sensitivity analyses (see Table 12 for a list of potential PRIMEtime CE sensitivity analyses).

**Table 11:** Values and settings used for PRIMEtime CE primary analyses

Variable	Value or setting
Annual discount rate for health outcomes	1.5%
Annual discount rate for costs	1.5%
Economic perspective	NHS England and social care costs for both modelled and unrelated diseases
Intervention costs	Costs to government and to industry where appropriate
Time horizon	10 years

**Table 12:** List of potential PRIMEtime CE sensitivity analyses

Sensitivity analysis	Explanation of what is changed compared to the primary analysis
Changing the time horizon.	Time horizon changing from 10 years in the primary analysis to 1 year, 5 years, 20 years, and 100 years (lifetime of the cohort).
Analysing results from an NHS perspective.	Estimating cost effectiveness using the change in NHS costs and intervention costs only (without any societal costs).
Analysing results from a social care perspective.	Estimating cost effectiveness using the change in social care and intervention costs only (without any NHS costs).
Including social care costs and productivity.	Adding an economic estimate of changes to productivity arising from the intervention.
Including all wider societal costs.	Including an economic estimate of the intervention on all wider societal costs (including productivity and social care costs).
Using a discount rate of 3.5%.	Changing the discount rate for costs and outcomes from 1.5 to 3.5%.
No disease costs estimated for diseases not explicitly modelled by PRIMEtime CE (unrelated disease costs).	Removing from the model any NHS and social care costs estimated to accrue due to diseases that are not explicitly modelled by PRIMEtime CE.
No cancer included in the model	Cancer removed from the model so that only IHD, stroke, type two diabetes, and liver cirrhosis are included.
Only including diseases directly related to the risk factor affected.	For a diet intervention, only IHD and stroke are included in the model, and for a physical activity intervention, IHD, stroke, type two diabetes, breast cancer, and colorectal cancer are included.

### Sensitivity analyses

Sensitivity analyses can be used with PRIMEtime CE to explore the effects on cost-effectiveness of changing various assumptions including time horizon, discount rate, and removing industry related costs (Table 9).

## Discussion

This paper describes the PRIMEtime CE model and its data inputs. PRIMEtime CE can help public health decision makers by estimating and directly comparing the cost effectiveness of interventions affecting population dietary habits and levels of physical activity, using the same underlying data and assumptions. Furthermore, PRIMEtime CE quantifies the health and social care costs - as well as any reduction in quality of life - arising as a consequence of developing diseases unrelated to those modelled. This makes it possible for decision makers to be reasonably confident (within the uncertainty intervals presented) that one intervention is likely to be more cost effective than another given the time horizon and economic perspective used.

### Addressing the challenges of public health economic modelling

PRIMEtime CE addresses some of the challenges of public health economic modelling, namely quantifying long term health outcomes, wider societal consequences, and the effects of multicomponent interventions [15, 17, 23].

Multistate life table models are well suited to modelling long terms health impacts and PRIMEtime CE can model outcomes over a population’s lifetime. Furthermore, both health and social care costs are included, thereby incorporating some wider societal consequences of the interventions modelled. There is a broader debate in the literature about how best to value outcomes from public health interventions where they may have societal and environmental costs and benefits beyond health that are not quantified using cost-utility analyses [14–18, 20, 81, 94, 95]. However, using cost utility analyses and reporting the cost per QALY is a useful method for health policy makers in England because it is consistent with the NICE approach to assessing medical interventions and - as things stand - is therefore relatively easy to interpret and compare with other health economic assessments [13].

A limitation of multistate life table models (and cohort models more generally) is that they are less flexible than some other modelling approaches (for example, using microsimulation) at modelling heterogeneous populations and quantifying the impact of an intervention on inequalities. In order to obtain results by population subgroup using PRIMEtime CE, the user would need to conduct multiple runs, each with a different population subgroup and using subgroup specific model input parameters.

Additionally, PRIMEtime CE does not fully address the challenges of modelling multicomponent interventions, for example, an intervention including both social marketing and legislative changes. Some more complex model structures, such as system dynamics models and discrete event simulation, might be better suited to this as they can simulate interactions between population subgroups, or between the population and the environment [24]. However, multistate life tables do allow for additional disease outcomes to be included without having to construct a new model meaning that they can be readily adapted if multicomponent interventions include diseases that are not already simulated. And PRIMEtime CE allows for the effect of an intervention on multiple different dietary and physical activity risk factors to be modelled simultaneously.

### Generalisability and comparisons with other models

It is intended that PRIMEtime CE will be used to produce comparable cost-effectiveness estimates of different public health policies, including ranking interventions, to help decision makers prioritise resources. Similar rankings have been compiled in the UK and elsewhere; for example, the NICE physical activity return on investment tool, [96, 97] the ACE Prevention programme of research in Australia, [98] and the BODE^3^ Programme in New Zealand [99]. The NICE physical activity return on investment tool compares how different physical activity interventions would affect health and costs for a population of interest. The tool is based on a Markov model that estimates how changes to levels of physical activity affect the prevalence of CHD, stroke, and type two diabetes. The tool has a user-friendly interface whereby various model inputs can be manipulated and the outputs analysed. Unlike PRIMEtime CE, however, the disease costs and utilities used by the model are from a variety of different sources and population groups.

The research programmes in Australia and New Zealand both used systematic and comparable approaches to estimating the cost-effectiveness of different public health interventions, with the explicit aim of informing policy. As with PRIMEtime CE, these were developed using country specific routine data where possible, with standardised approaches to estimating utilities and costs. The BODE^3^ programme of research derived age, sex, and disease specific health costs from Health Tracker - national data that individually links costs with health events, [100] and New Zealand specific disability weights are taken from the Global Burden of Disease study [101]. The ACE programme used the DISMOD tool and data from the Netherlands to estimate disability weights based on Australian burden of disease data (see Begg et al. for details [102]), and costs were taken from a national dataset with disease specific healthcare cost estimates [103]. The use of country specific data sources mean that although results from the ACE and BODE^3^ programmes of research may not be directly applicable to England, both can rank interventions.

### Strengths and limitations

PRIMEtime CE has several strengths. Bias is minimised by adopting a consistent and systematic approach to identifying model input parameters, in particular disease costs and utilities; model uncertainty is quantified; there are options for multiple sensitivity analyses; and changes to costs and quality of life from diseases unrelated to those modelled can be estimated.

There are some important methodological limitations of both the data sources used by PRIMEtime CE, and the model’s structure. Firstly, multistate life table models assume diseases are independent of one another. This means that the proportion of the cohort existing in one disease state does not affect the probability of the cohort developing any other disease states - the model cannot distinguish between whether the population with breast cancer is the same as that with heart disease. Multistate life table models may therefore over- or under-estimate utilities and costs due to differences occurring between two diseases existing in two separate individuals or being co-morbid within the same individual. To help counter this limitation, PRIMEtime CE has been developed such that there is a dynamic relationship between type two diabetes and cardiovascular disease (an increase in diabetes prevalence increases the risk of the modelled cohort developing IHD and stroke, and the relative risk of IHD and stroke incidence due to changes in BMI or physical activity is adjusted to prevent double counting due to a concomitant rise in diabetes). However, this adjustment does not occur for other diseases which, depending on the intervention simulated, may over- or under-estimate the resulting cost per QALY. Secondly, no interactions between individuals within the population or between the population and its environment are simulated. Including these interactions requires data on their effects and would generate more uncertainty in model results, but conversely it may also mean the model better represents reality and is more accurate. Thirdly, PRIMEtime CE only models the effect of changing a risk factor on disease incidence and not case fatality rates. If simulated interventions lead to reductions in case fatality rates, the model may under-estimate health and cost outcomes arising from increased longevity (both for modelled and unrelated diseases).

Further limitations are introduced through using NHS England programme budgeting data to estimate disease costs where miscoding of NHS England expenditure may mean that some annual disease costs are overestimated, and others underestimated. It is not possible to quantify any misallocation of costs, however the effect on outcomes will be limited by the fact that the majority - 78% - of the NHS England budget is accounted for through disease specific programme budget categories. Disease costs are also only estimated per prevalent case whereas in reality costs vary by time since diagnosis and proximity to end of life. This is a limitation of using a top-down method for estimating costs that may over- or under-estimate total costs, particularly where there are changes to case fatality rates. Further details on the limitations of the costing methods, as well as the PRIMEtime model and social care costing can be found elsewhere [31, 79, 85].

Finally, there are limitations of the utility decrements estimated by Sullivan et al [80]. In particular, EQ-5D data are sampled from a 2000–2003 US population rather than a more recent UK population; and individuals at very early or late stages of their disease are likely to be under-represented due to being too unwell or unwilling to participate meaning disease specific utility decrements might under-estimate the true average reduction in quality of life. The systematic review of breast cancer utilities provides some reassurance that the values reported by Sullivan et al. are reasonable estimates. Furthermore, baseline age and sex utility values are derived based on all diseases in the population rather than only unrelated diseases (those not explicitly modelled by PRIMEtime CE). Therefore, utility decrements that accrue due to increased longevity following an intervention may over-estimate the true utility decrement. However, the effect of this on model results is limited due to discounting and the relatively smaller proportion of the modelled cohort living to old age.

Future intended developments of PRIMEtime CE are to include further testing of the model’s validity [93] and to expand PRIMEtime CE’s scope. This may include consideration of how the model can be used to quantify the effects of policies on inequalities; the addition of further behavioural risk factors such as alcohol and tobacco, and the use of drugs in primary prevention; how the model can be made more user-friendly by the development of non-technical interface; and whether results can be quantified at the local authority level.

## Conclusions

In this paper we describe PRIMEtime CE, a model that allows public health policies affecting multiple diseases to be directly compared with one another through using the same methods to estimate the effect of changing a risk factor on a disease outcome, disease costs, and disease morbidity. We intend that future work will expand the model’s policy relevance through developing the range of risk factors and outcomes simulated.

## Additional file

Additional file 1: Results of the validation of Sullivan et al. utility values, and protocol for systematic review of breast cancer utility values. (DOCX 67 kb)

## Data Availability

Datasets used and analysed in this study are publicly available and are either reported in the additional data file or are listed with appropriate references. This with the exception of specialised services expenditure data supplied to the corresponding author by NHS England which the corresponding author does not have permission to share. These data can be made available with permission from both the corresponding author and NHS England. All other data used or generated by the study are available from the corresponding author on request.

## Abbreviations

APS: Active People Survey
BMI: Body mass index
HTA: Health technology assessment
IHD: Ischaemic heart disease
ISPOR-SMDM: International Society for Pharmacoeconomics and Outcomes Research-Society for Medical Decision Making
MET: Metabolic equivalent of task
NCDs: Non-communicable diseases
NDNS: National Diet and Nutrition Survey
NHS: National Health Service
NICE: National Institute for Health and Care Excellence
QALY: Quality adjusted life year
UI: Uncertainty interval
WHO: World Health Organization
