# Supplementary material for: PRIMEtime CE: a multistate life table model for estimating the cost-effectiveness of interventions affecting diet and physical activity
Source: BMC Health Serv Res. 2019 Jul 16;19:485. doi: 10.1186/s12913-019-4237-4 (PMC6633614; doi:10.1186/s12913-019-4237-4)
Supplement: Supplementary file 1 — Results of the validation of Sullivan et al. utility values, and protocol for systematic review of breast cancer utility values. (DOCX 67 kb) [file 12913_2019_4237_MOESM1_ESM.docx]

**Additional data file**

A D M Briggs, L Cobiac, J Wolstenholme, P Scarborough. PRIMEtime CE: a multistate life table model for estimating the cost-effectiveness of interventions affecting diet and physical activity.

**Contents**

Results of the validation of Sullivan et al. utility values………………….………………………..……………..2

## Protocol for systematic review of breast cancer utility values………………….……………..………………4

**References**………………………………………………………………………………………………………………………….…..12

Results of the validation of Sullivan et al. utility values

Table S1. Comparison of breast cancer utility values identified through the systematic review and by Sullivan et al.[1]

| Study | Patient population | Range of published utilities (SE/95% confidence intervals) |
| --- | --- | --- |
| Sullivan et al., 2011[1] | Catalogue of EQ-5D scores for the United Kingdom (EQ-5D results from US population, valued by a UK population). Patients with ICD-9 code 174, malignant neoplasm female breast | 0.75 (0.72-0.78) |
| Prescott et al., 2007[2] | English and Scottish patients with surgically treated node negative breast cancer up to 15 months post treatment with or without radiotherapy | 0.72 (0.68-0.76) to  0.78 (0.74-0.81) |
| Hall et al., 2015[3] | English breast cancer survivors up to 15 months post diagnosis | 0.74 (0.71-0.78) to  0.83 (0.76-0.89) |
| Haines et al., 2010[4] | Australian newly diagnosed patient undergoing adjuvant chemotherapy with or without a home-based exercise programme | 0.78 (0.19) to 0.85 (0.19) |
| Vrettos et al., 2012[5] | Greek breast cancer patients presenting to an oncology day clinic | 0.69 (0.29) |

## Protocol for systematic review of breast cancer utility values

This protocol describes how to systematically identify breast cancer utility values from the literature, using the method described by Papaioannou et al.[6]

1. Search strategy
2. Inclusion criteria

- Paper describes empirically estimated original health state utility value or is a systematic review of utility values
- Utility value relates to health state of interest
- EQ-5D used to describe health state
- Patient population used to measure change in HRQoL
- Description given of technique used to value health state and of population used to value change to health related quality of life (HRQoL)
- Study population aged > 17 years of age and applicable to the English population suffering from disease (applicable to the English population is defined as studies with patients from Europe, United States of America, Canada, Australia, and New Zealand)
- Published in English

1. Databases

- MEDLINE
- EMBASE
- PsychINFO
- Science Citation Index (ISI)
- Social Sciences Citation Index
- Conference Proceedings Citation Index – Science (CPCI-S)
- Cochrane library (CDSR, CENTRAL, DARE, CMR, HTAD, NHSEED, ABOUT)
- The EQ-5D website
- (The Cost-Effectiveness Analysis Registry at Tufts Medical Centre not searched because of not having premium access thereby limiting search options and results)

1. Supplementary searching

Search of reference lists (including of non-included reviews)

1. Search terms

Search terms for identifying breast cancer utility values developed using those found in Papaioannou et al. 2010 and Peasgood et al. 2010 [6, 7].

An example of search terms used is shown below for Web of Science:

Indexes=SCI-EXPANDED, SSCI, CPCI-S Timespan=1945-2015

1. TS=(euroqol OR euro qol OR eq5d OR eq 5d)
2. TS=((breast NEAR/2 cancer*) OR (breast NEAR/2 neoplasm*) OR (breast NEAR/2 carcinoma*) OR (breast NEAR/2 neoplasm*) OR (breast NEAR/2 tumo*) OR (malignan* NEAR/2 breast) OR (mammary NEAR/2 cancer*) OR (mammary NEAR/2 neoplasm*) OR (mammary NEAR/2 carcinoma*) OR (mammary NEAR/2 neoplasm*) OR (mammary NEAR/2 tumo*) OR (malignan* NEAR/2 mammary))
3. #1 AND #2
4. Refining the search

The search strategy can iteratively develop to balance the sensitivity and specificity of the search.

1. Deciding which utility values to report
   1. Flow chart for deciding which utility values to report is shown in figure S1.

**Figure S1.** Flow chart for deciding which utility values to report

More than one appropriate utility value available?

Redefine health state to be more generic and repeat search

Report utility value

Report range of utility values

answer yes

answer no/unknown

Utility value available that meets inclusion criteria

Utility values score differently on quality assessment

Report utility value with highest quality assessment score

- 1. Study quality assessment

If a systematic review needs to be quality assessed, use adapted Critical Appraisal Skills Programme (CASP) checklist (box S1).[8] For the systematic review to be considered good quality, the answer to questions 1-5 and to question 9 in box S1 should all be *yes*.

**Box S1.** Adapted CASP-style checklist for appraising the quality of a systematic review of utility values[6, 8]

| For the following questions, answer Yes, No, Partly, or Can’t tell:  **1. Did the review ask a clearly-focused question?**  Consider if the question is focused in terms of:   - Population describing the health states (ideally patients) - Population valuing the change in HRQoL (ideally public) - Method of elicitation (ideally choice-based method e.g. TTO)   **2. Did the review include the right type of study?**  Consider if the included studies:   - address the review’s question. - are appropriate studies.   **3. Did the reviewers try to identify all relevant studies?**  Consider as a minimum:   - Were a number of electronic databases searched? (ideally clinical and specific health economic) - Were reference lists scrutinised for retrieved references?   Ideally, but not mandatory, consider that the search methods should involve:   - - personal contact with experts.   - searches for unpublished studies.   - citation and author searching.   **4. Did the reviewers assess the quality of the included studies?**  Consider the:   - Sample size. - Respondent selection and recruitment. - Inclusion/exclusion criteria. - Response rates to instrument used. - Numbers (%) lost to follow-up. - Are reasons provided for any loss to follow-up? - How is missing data from the instruments used to describe the health states dealt with? Is the method rigorous? - Any other problems with the study.   **5.** **Did the reviewers assess the relevance of the included studies to the review question?**   - Population describing the health states (ideally patients). - Population valuing the HRQoL (ideally public). - Method of elicitation (ideally choice-based method e.g. TTO).   **6.** **If the results of the studies have been combined, was it reasonable to do so?**   - Are the results of each study are clearly displayed? - Are the results were similar from study to study (look for tests of heterogeneity)? - Are the reasons for any variations in results are discussed?   **7. How are the results presented and what is the main result?**   - Is there a full account of why studies were excluded? - Is there are full justification of why studies were included? - How are the results expressed (descriptive statistics or coefficients of a model)?   **8. How precise are these results?**  Consider:   - - if a confidence interval was reported, would your decision about whether or not to use this intervention be the same at the upper confidence limit as at the lower confidence limit?   - if a p-value is reported where confidence intervals are unavailable.   **9. Can the health state utility values be used in the health states in your decision model?**  **Consider:**   - how relevant the population describing the health state is to the health states in the decision model. - have all subgroups been considered e.g. age, disease severity, setting? - do the utility values match the NICE reference case? - how do the results need to be modified for the decision model? |
| --- |

If assessing empirical studies, the criteria in table S2 will be used to assess study quality and applicability to the English setting, adapted from Papaioannou et al. 2010.[6] No study will be excluded on the basis of the score obtained, instead the score will be used to help decide which utility values to report (see figure S1). If comparing a review with an empirical study, a judgement based on the two separate quality assessment tools will be used to decide which utility value to report.

**Table S2.** Assessing quality and applicability of empirical studies estimating utility values

| Criteria | What to consider | Yes/partially/no (score 2/1/0) |
| --- | --- | --- |
| Uncertainty | Does the estimated utility value include the variance reflecting the sample size? |  |
| Respondents | Is the study population comparable to that in the model (consider inclusion criteria)? |  |
| Response rates | Is response rate reported and large enough for it not to bias results? |  |
| Loss to follow-up and missing data | Are losses to follow-up and missing data small and unlikely to lead to bias? |  |
| Instrument used | Is EQ-5D used to describe health states? |  |
| Population using instrument | Did the relevant patient population describe the change in health state and is the patient population applicable to the English setting (score 2 if UK patient population, score 1 if non-UK patient population but applicable to UK*)? |  |
| Population valuing change in HRQoL | Was the valuation of change in health state completed by the UK (score 2) or UK applicable (score 1) general population? |  |
| Technique of valuing health state | Was TTO used (score 2), or a different choice based method (score 1)? |  |
| Any other problems | Deduct 0, 1, or 2 points depending on number and scale of problems |  |

*countries applicable to UK are defined as countries in Europe as well as the United States of America, Canada, Australia, and New Zealand.

**References**

1. Sullivan PW, Slejko JF, Sculpher MJ, Ghushchyan V. Catalogue of EQ-5D scores for the United Kingdom. Med Decis Making. 2011;31:800–4. doi:10.1177/0272989X11401031.

2. Prescott RJ, Kunkler IH, Williams LJ, King CC, Jack W, van der Pol M, et al. A randomised controlled trial of postoperative radiotherapy following breast-conserving surgery in a minimum-risk older population. The PRIME trial. Health Technol Assess. 2007;11:1–149, iii–iv.

3. Hall PS, Hamilton P, Hulme CT, Meads DM, Jones H, Newsham A, et al. Costs of cancer care for use in economic evaluation: a UK analysis of patient-level routine health system data. Br J Cancer. 2015;112:948–56. doi:10.1038/bjc.2014.644.

4. Haines TP, Sinnamon P, Wetzig NG, Lehman M, Walpole E, Pratt T, et al. Multimodal exercise improves quality of life of women being treated for breast cancer, but at what cost? Randomized trial with economic evaluation. Breast Cancer Res Treat. 2010;124:163–75. doi:10.1007/s10549-010-1126-2.

5. Vrettos I, Kamposioras K, Kontodimopoulos N, Pappa E, Georgiadou E, Haritos D, et al. Comparing health-related quality of life of cancer patients under chemotherapy and of their caregivers. ScientificWorldJournal. 2012;2012:135283. doi:10.1100/2012/135283.

6. Papaioannou D, Brazier J, Paisley S. NICE DSU technical support document 9: The identification, review and synthesis of health state utility values from the literature. Sheffield; 2010.

7. Peasgood T, Ward SE, Brazier J. Health-state utility values in breast cancer. Expert Rev Pharmacoecon Outcomes Res. 2010;10:553–66. doi:10.1586/erp.10.65.

8. Critical Appraisal Skills Programme. CASP Economic Evaluation Checklist. Oxford; 2013. http://media.wix.com/ugd/dded87_3b2bd5743feb4b1aaac6ebdd68771d3f.pdf.
